# Improvement in Glycolipid Metabolism Parameters After Supplementing Fish Oil-Derived Omega-3 Fatty Acids Is Associated with Gut Microbiota and Lipid Metabolites in Type 2 Diabetes Mellitus

**DOI:** 10.3390/nu16213755

**Published:** 2024-10-31

**Authors:** Jiayue Xia, Shiyu Yin, Junhui Yu, Jiongnan Wang, Xingyi Jin, Yuanyuan Wang, Hechun Liu, Guiju Sun

**Affiliations:** 1 Key Laboratory of Environmental Medicine and Engineering of Ministry of Education, School of Public Health, Southeast University, Nanjing 210009, China; 230229010@seu.edu.cn (J.X.); yinshiyu@seu.edu.cn (S.Y.); jshmyjh@126.com (J.Y.); 230239098@seu.edu.cn (J.W.); xingyijin@foxmail.com (X.J.); 230218460@seu.edu.cn (Y.W.); 2 Department of Nutrition and Food Hygiene, School of Public Health, Southeast University, Nanjing 210009, China; 3 Department of Endocrinology and Metabolism, The First Affiliated Hospital of Nanjing Medical University, 300 Guangzhou Road, Nanjing 210029, China

**Keywords:** omega-3 fatty acids, blood lipids, blood glucose, gut microbiota, glycerophospholipid, type 2 diabetes mellitus

## Abstract

Background/Objectives: This study aimed to investigate the effects of fish oil-derived omega-3 polyunsaturated fatty acids (omega-3 PUFAs) on gut microbiota and serum lipid metabolites in T2DM. Methods: In a three-month, randomized, double-blind, placebo-controlled study, 110 T2DM patients received either fish oil (*n* = 55) or corn oil (*n* = 55) capsules daily. Serum lipids, glycemic parameters, gut microbiota diversity, and lipidomics were assessed. Results: This study found that fish oil-derived omega-3 PUFAs intervention did not significantly lower the fasting plasma glucose levels when compared with the baseline level (*p* > 0.05). However, serum fasting blood glucose (*p* = 0.039), glycosylated hemoglobin levels (*p* = 0.048), HOMA-IR (*p* = 0.022), total cholesterol (*p* < 0.001), triglyceride (*p* = 0.034), LDL cholesterol (*p* = 0.048), and non-HDL levels (*p* = 0.046) were significantly lower in the fish oil group compared with the corn oil group after three months of intervention. Also, it altered glycerophospholipid metabolism and gut microbiota. After three months, the fish oil group showed a significantly lower abundance of *Desulfobacterota* compared with the corn oil control group (*p* = 0.003), with reduced levels of *Colidextribacter* (*p* = 0.002), *Ralstonia* (*p* = 0.021), and *Klebsiella* (*p* = 0.013). Conversely, the abundance of *Limosilactobacillus* (*p* = 0.017), *Lactobacillus* (*p* = 0.011), and *Haemophilus* (*p* = 0.018) increased significantly. In addition, relevant glycolipid metabolism indicators showed significant correlations with the altered profiles of serum lipid metabolites, intestinal bacteria, and fungi. Conclusions: This study highlights the impact of fish oil-derived omega-3 PUFAs on intestinal microbiota structure and function in patients with type 2 diabetes. The observed decrease in pathogenic bacterial species and the enhancement of beneficial species may have significant implications for gut health and systemic inflammation, both of which are pivotal in managing diabetes. Further research is warranted to comprehensively elucidate the long-term benefits and underlying mechanisms of these microbiota alterations.

## 1. Introduction

Type 2 diabetes mellitus (T2DM) is a prevalent chronic metabolic condition, affecting more than 90% of individuals with diabetes. According to data from the International Diabetes Federation, the worldwide prevalence of diabetes among individuals aged 20–79 years is expected to reach 12.2% (783.2 million people) by 2045 [1]. The progression of T2DM can lead to multi-organ damage and complications such as diabetic foot, diabetic neuropathy, and diabetic nephropathy [2]. The prevalence of type 2 diabetes is associated with a variety of risk factors, including genetic factors, advancing age, racial differences, and lifestyle choices. The waist-to-hip ratio (WHR) is a valuable metric for evaluating abdominal obesity, a significant risk factor for developing type 2 diabetes. Individuals exhibiting an elevated waist-to-hip ratio are at an increased risk of developing insulin resistance, a significant risk factor for type 2 diabetes. WHR provides information beyond BMI to predict diabetes risk more accurately. Therefore, monitoring and controlling the waist-to-hip ratio is also an important health management measure for individuals with type 2 diabetes or who are at risk for diabetes. Weight loss and lifestyle modifications are effective in treating T2DM, but patient acceptance and adherence are low. Various synthetic drugs with antidiabetic effects are currently being tested in clinical trials. However, these drugs have multiple undesirable effects, such as increased weight, low blood sugar, fluid retention, and heart failure. In this regard, the quest for a dietary supplement that is efficacious without inducing side effects could offer a novel approach for adjunctive therapy in T2DM.

Omega-3 polyunsaturated fatty acids (omega-3 PUFAs) mainly include α-linolenic acid, eicosapentaenoic acid (EPA), and docosahexaenoic acid (DHA) [3]. Omega-3 PUFAs have multiple health benefits for cardiovascular disease (atrial fibrillation, atherosclerosis, thrombosis, inflammation, and sudden cardiac death), diabetes, cancer, depression and various psychiatric disorders, age-related cognitive impairment, periodontal disease, and rheumatoid arthritis [4]. Several studies have revealed that omega-3 PUFAs have beneficial effects on preventing T2DM through a variety of mechanisms, including insulin signaling, anti-inflammatory effects, and the expression of genes that control glucose metabolism [5,6]. Some studies in adults have found that the intestinal microbiota changes following omega-3 PUFAs supplementation. The omega-3 PUFAs may improve these diseases by restoring the microbiota composition and increasing the production of anti-inflammatory compounds, like short-chain fatty acids [7]. Disruption of the intestinal microbiota balance results in decreased levels of related biochemical factors, short-chain fatty acids, bile acids, and endocrine regulatory peptides, as well as increased levels of lipopolysaccharides, ultimately leading to insulin resistance and increasing the risk of T2DM [8]. A well-balanced intestinal flora can stimulate the body’s metabolism and strengthen the immune function. Regulation of intestinal flora can improve insulin resistance, elevate insulin levels, and regulate blood glucose levels.

Triglycerides contain many individual molecular species, whereas lipoproteins contain many different lipid classes that contain multiple molecular species [9]. The role of these individual molecular lipid species in the development of T2DM remains unclear. A study conducted in 2018 found that baseline lysophospholipids (LPs), including lysophosphatidylcholines (LPCs) and lysophosphatidylethanolamines (LPEs) and phosphatidylcholine-plasmalogens (PC-PLs) were negatively related to T2DM risk, while baseline triacylglycerols (TAGs) and diacylglycerols (DAGs) were directly associated with T2DM incidence [10]. Additionally, toxic 1-deoxyceramides and etheric lipids are hallmarks of both lean and obese type 2 diabetics [11]. A study found that diabetic polyneuropathy in people with type 2 diabetes is associated with changes in plasma metabolites [12]. The findings suggest that dysfunctional lipid metabolism is closely related to diabetes development, and future directions could focus on examining exogenous as well as endogenous factors that regulate lipid pathways and testing to identify potential targets for the treatment of T2DM.

Omega-3 PUFAs can regulate the diversity and abundance of intestinal flora. Supplementation of perilla oil rich in linolenic acid improves intestinal dysbiosis in diabetic mice by decreasing the abundance of the *Blautia* genus [13]. Results from dietary surveys indicated a significant association between the level of total omega-3 PUFAs and the alpha diversity of the gut microbiome (Shannon index) [14]. Nevertheless, there is still variation in the results of studies on gut bacteria, and it remains uncertain whether omega-3 PUFAs can also influence the role of intestinal fungi among T2DM. Furthermore, the main lipid metabolites of omega-3 PUFAs that influence T2DM are still uncertain and warrant further investigation. Therefore, in this study, we aimed to explore the protective effect of fish oil-derived omega-3 PUFAs supplementation and its underlying mechanism using 16S ribosomal RNA (rRNA) sequencing of the gut microbiome and untargeted lipid metabolomics of serum.

## 2. Materials and Methods

### 2.1. Patient Recruitment and Ethics Approval

Subjects with T2DM were recruited in Huai’an, China, from June to 1 August 2019. The inclusion criteria for this study were males or females aged 18–70 years old with diagnosed type 2 diabetes mellitus [15,16]. In addition, the patients with type 2 diabetes included were individuals that were very well controlled by their medication. The exclusion criteria were women who were pregnant or breastfeeding, patients with poorly controlled diabetic conditions, patients with severe diabetic complications, patients with severe immune system disorders, patients who had taken fish oil-related supplements within the last six months, patients with special dietary habits (such as vegetarians, weight managers, or ketogenic dieters), or patients with hepatic or renal insufficiency, malignant tumors, and hormone therapy.

This randomized, double-blinded, placebo-controlled trial was recorded in the Chinese Clinical Trial Registry at https://www.chictr.org.cn/showproj.html?proj=44628 (accessed on 2 December 2019). This study was conducted according to the guidelines laid down in the Declaration of Helsinki as revised in 2013; all procedures that involved human subjects were approved by the ethical review of scientific research projects by Lian Shui People’s Hospital, affiliated with Kang-da College of Nanjing Medical University (NO. 2019710-6), and all participants provided written informed consent.

### 2.2. Study Design and Intervention

This study was a three-month, randomized, double-blind, and placebo-controlled clinical trial. Participants with T2DM (*n* = 110) were randomly allocated to receive three grams of fish oil capsules (*n* = 55) or corn oil capsules (*n* = 55) per day. Fish oil supplement capsules contained omega-3 PUFAs supplied by the Royal DSM Group, the Netherlands. Each 100 g fish oil capsule contains mainly 36.86 g of EPA, 17.47 g of DHA, and other types of fatty acids (Royal DSM, Heerlen, The Netherlands). The corn oil group was used as the control group. Each 100 g corn oil capsule contained 45.64 g of linoleic acid, 25.62 g of oleic acid, 17.0 g of palmitic acid, and other fatty acids (Jiangsu Nature’s Way Bio-technology Co., Yixing in Jiangsu, China); participants were required to take 3 g of fish oil capsules or corn oil capsules per day. The capsules in the two groups were similarly shaped, sized, and colored. Test samples were stored in a dry, light-proof environment at a room temperature of 25 °C.

### 2.3. Clinical Measures

Blood samples from all participants at baseline and intervention endpoints were collected for the testing of lipids (including total cholesterol, triglycerides, low-density lipoprotein cholesterol, and high-density lipoprotein cholesterol) and glycemic markers (including fasting blood glucose, glycosylated hemoglobin, and fasting insulin). The serum samples and feces of all participants were used to detect the intestinal flora and lipid metabolites. In addition, we collected information about the patients with type 2 diabetes, including their age, education, duration of T2DM, smoking habits, physical activity, and medications. All participants were counseled by a physician at baseline on the dietary habits and were required to follow the recommended diet during the intervention.

### 2.4. Blood Sampling, Feces Sampling, and Analytical Methods

All samples were collected with the informed consent of the patient. The serum from fasting (9 h minimum) blood samples was separated and frozen at −80 °C before being analyzed by quantitative lipidomics. The liquid chromatography–mass spectrometry (LC-MS/MS) metabolomic analysis of serum lipids was performed by Biotree (Shanghai, China). The detailed metabolite extraction procedure was as follows: (1) add 500 μL of extraction solution to an appropriate amount of blood sample; (2) vortex and mix, centrifuge the sample at 4 °C for 15 min after sonication, remove the supernatant, and vacuum dry; (3) add 100 μL of resolvent to the dried sample and vortex and sonicate; (4) centrifuge the sample at 4 °C for 15 min; (5) take the supernatant in the injection bottle and perform an assay; (6) take another 10 μL of supernatant from all samples and mix them into quality control samples for the assay. The lipidomics onboard assay was performed mainly using a SCIEX ExionLC ultra-high performance liquid chromatograph (CL Technology Co., Ltd., New Taipei City, Taiwan, China) with a liquid chromatographic column for the color separation of target compounds [17,18]. The chromatographic separation was performed on a liquid chromatographic column. The liquid chromatography phase A was 60% acetonitrile, and phase B was 10% acetonitrile. Column temperature: 40 °C; sample tray temperature: 6 °C; injection volume: 2 μL; sample volume: 2 μL. All quantitative analyses of the target compounds were performed by Skyline software (version 23.1). The absolute content of each lipid was calculated according to the internal standard method. In addition, 16S ribosomal RNA (rRNA) sequencing of the V1–V9 region and the fungal diversity analysis of internally transcribed spacer sequences (ITSs) were performed to detect the gut microbiota composition and diversity of all fecal samples.

### 2.5. Statistical Analysis

The distribution of the data was analyzed by visual inspection of the histogram, and as most of the variables were not normally distributed, a nonparametric test was applied. Categorical variables were tested using chi-square tests. Data for quantitative lipidomics were scaled and logarithmically transformed to reduce the impact of noise and high variance. A PCA analysis (principal component analysis, PCA) was then performed to visualize the distribution and grouping of the samples after these transformations. A supervised orthogonal projection to latent structures with discriminate analysis (OPLS-DA) was used to visualize group separation and identify significantly changed metabolites. In the OPLS-DA analysis, the variable importance in projection (VIP) of the first principal component was obtained. Metabolites with VIP > 2, fold change > 2, and *p* < 0.05 were considered as significantly changed metabolites. To examine the relationship between microbiota and lipid metabolites, a Spearman correlation was conducted within groups. SPSS (version 26.0, IBM, Armonk, NY, USA) and RStudio software (version 2024.09.0) were used for all correlation tests.

## 3. Results

### 3.1. Demographic and Clinical Characteristics

**Figure 1:**
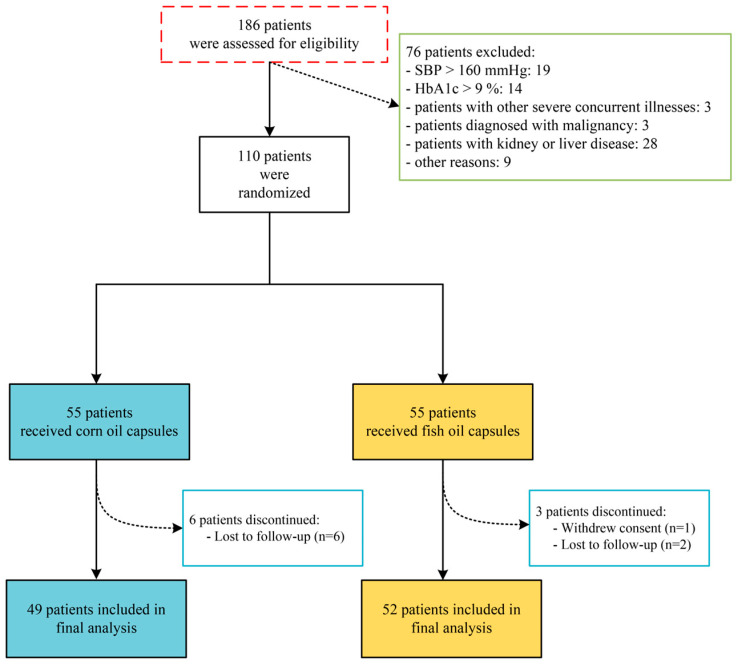
Study flowchart. SBP, systolic blood pressure.

A total of 186 volunteers completed the initial screening physical examination, of which 19 had systolic blood pressure higher than 160 mmHg, 14 had glycosylated hemoglobin greater than 9%, 1 had severe diabetic foot disease, 1 had severe heart disease, 4 had severe thyroid disease, 3 had malignant tumor patients, 14 had kidney disease, 14 had liver disease, and 9 were excluded for other reasons. A total of 110 volunteers were divided into fish oil and corn oil groups according to the randomized grouping method, with 55 volunteers in each group. After three months of intervention, a total of 101 people eventually completed the trial, including 52 in the fish oil group and 49 in the corn oil group. The details are shown in Figure 1.

As shown in Table 1, the mean age of the study subjects was 53.81 years, the mean age of the corn oil group was 54.29 years, and the mean age of the fish oil group was 53.37 years. The study subjects were more predominantly male. The median duration of diabetes was 6 years, and diabetes was treated mainly with oral hypoglycemic drugs or oral hypoglycemic drugs combined with annotated insulin, while a small number used simple dietary control without taking hypoglycemic drugs or insulin injections. The diabetes mellitus of the study participants was very well controlled by their medication. The mean waist-to-hip ratio of the study subjects was 0.91. In terms of sex, age, diabetes duration, diabetes treatment, waist-to-hip ratios, and systolic and diastolic blood pressures, there were no significant differences between the two groups. In addition, there were no significant differences in the total dietary intake of energy, dietary intake of protein, amount of carbohydrates, amount of fat, and fiber in the corn oil and fish oil groups before and after the intervention (Table 2).

### 3.2. Impact of Omega-3 PUFAs Intervention on Glucose Parameters and Lipid Profiles in Patients with T2DM

As shown in Table 3, after three months of intervention, serum fasting blood glucose was significantly higher in the corn oil group (*p* < 0.001), and there was no significant change in the fish oil intervention group when compared with the baseline level (*p* = 0.328). In addition, serum fasting blood glucose (*p* = 0.039), glycosylated hemoglobin levels (*p* = 0.048), and HOMA-IR (*p* = 0.022) were significantly lower in the fish oil intervention group than in the corn oil control group at three months. Serum insulin levels were significantly lower in the fish oil group after three months of intervention compared with the baseline level (*p* = 0.019). Overall, fish oil-derived omega-3 PUFAs supplementation did not significantly lower the fasting plasma glucose levels (*p* > 0.05). Also, this study observed a significant deterioration in fasting blood glucose and HOMA-IR levels in the corn oil control group, suggesting that the insulin sensitivity of participants became worse in the corn oil control group.

In terms of lipid profiles, the fish oil intervention group had significantly lower serum triglyceride (*p* = 0.001), total cholesterol (*p* < 0.001), and non-HDL levels (*p* < 0.001) and significantly higher HDL-C levels (*p* < 0.001) compared with the baseline level after three months of intervention. Total cholesterol (*p* < 0.001), triglyceride (*p* = 0.034), LDL cholesterol (*p* = 0.048), and non-HDL levels (*p* = 0.046) were significantly lower in the fish oil intervention group compared with the corn oil control group after three months of intervention.

### 3.3. Impact of Omega-3 PUFAs Intervention on Gut Microbiota and Fungi Richness, Diversity, and Composition in Subjects with T2DM

**Figure 2:**
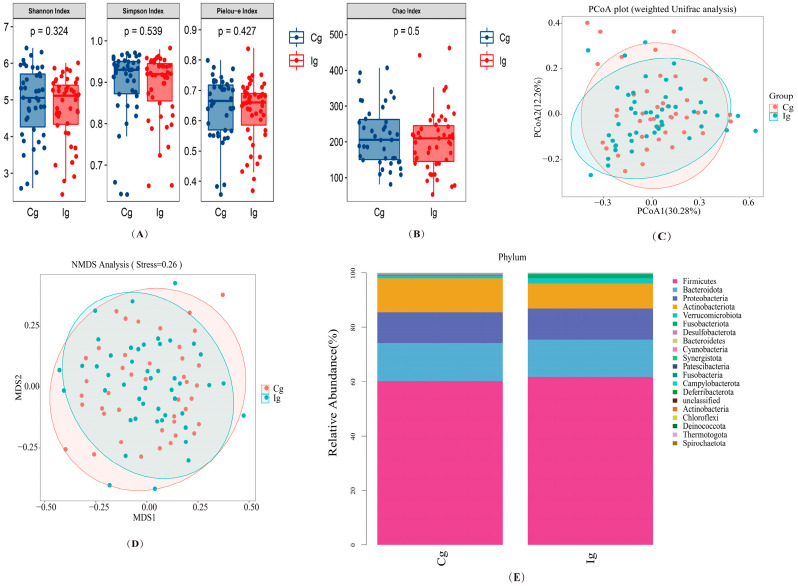
The effect of omega-3 PUFAs on the diversity of gut microbiota and compositions at the phylum. (**A**,**B**) α-diversity indices as reflected by the Shannon, Simpson, Pielou-e estimator, and Chao1 indices. *p* values were calculated using Mann–Whitney tests. (**C**) PCoA score plot based on a weighted UniFrac analysis for all participants. (**D**) β-diversity measured via NMDS. (**E**) Phylum level of gut bacteria. Only taxonomic groups that are 1% or greater are shown. Cg, corn oil group; Ig, fish oil group.

A total of 4,579,215 quality reads of 89 samples were generated with an average of 51,454 ± 8885 reads per sample for gut bacteria. In addition, a total of 5,701,798 quality reads of 87 samples were generated with an average of 65,538 ± 11,843 reads per sample for the gut fungal community. The alpha diversity of gut bacterial communities revealed that no significant differences were found for the Chao1, Shannon, Simpson, and Pielou-e estimator between two groups (Figure 2A,B).

As shown in Figure 2C, there was no statistically significant variation in clustering between the two groups. Similarly, the β-diversity measured using non-metric multi-dimensional scaling (NMDS) based on the Bray–Curtis dissimilarity index also showed no significant difference (Figure 2D).

Differences in the relative abundance of the gut microbiota between the two groups at the phylum level are represented as stacked histograms. Figure 2E illustrates the overall microbial compositions between two groups after three months. For the fish oil group, *Firmicutes*, *Bacteroidetes*, and *Proteobacteria* account for about 73.2% of the total at the phylum level of gut bacteria. The *Firmicutes*/*Bacteroidetes* ratio (F/B) is an indicator of intestinal dysbiosis and obesity. This ratio tends to increase after fish oil intervention. However, no significant changes were observed in the relative abundance of *Bacteroidetes* and *Proteobacteria* after fish oil intervention.

**Figure 3:**
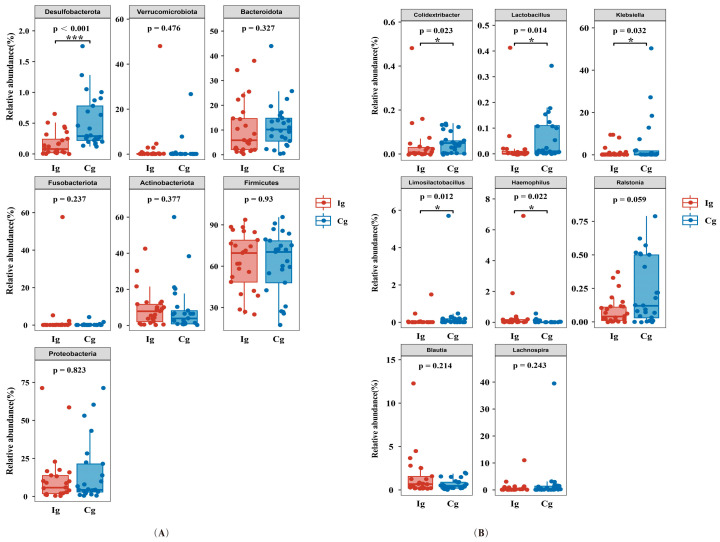
Changes in the relative abundance of gut bacteria after intervention. (**A**) Phylum level of gut bacteria; (**B**) genus level of gut bacteria. * *p* < 0.05, *** *p* < 0.001; Cg, corn oil group; Ig, fish oil group. FDR < 0.2 was considered as significant.

Changes in the relative abundance of gut bacteria after intervention at the phylum level and genus level are presented in Figure 3A,B. At the level of the intestinal bacterial phylum, the fish oil group had a significantly lower relative abundance of *Desulfobacterota* than the corn oil control group after three months (*p* = 0.003). At the level of intestinal bacterial genera, the relative abundance of *Colidextribacter* (*p* = 0.002), *Ralstonia* (*p* = 0.021), and *Klebsiella* (*p* = 0.013) were significantly lower in the fish oil group when compared with the corn oil control group after three months. The relative abundance of *Limosilactobacillus* (*p* = 0.017), Lactobacillus (*p* = 0.011), and *Haemophilus* (*p* = 0.018) were significantly higher in the fish oil group compared with the corn oil control group after three months.

**Figure 4:**
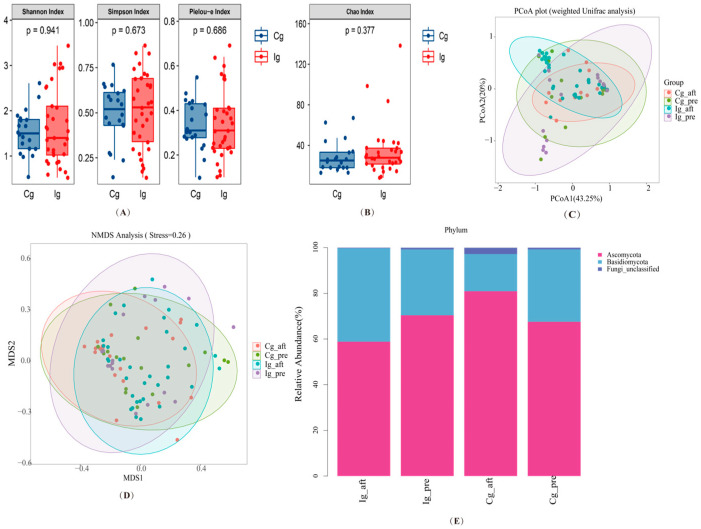
The effect of omega-3 PUFAs on the diversity of gut fungi at the phylum. (**A**,**B**) α-diversity indices as reflected by the Shannon, Simpson, Pielou-e estimator, and Chao1 indices at the end of intervention. *p* values were calculated using Mann–Whitney tests. (**C**) PCoA score plot based on weighted UniFrac analysis for all participants. (**D**) β-diversity measured via NMDS. (**E**) Phylum level of gut fungi. Only taxonomic groups that are 1% or greater are shown. Cg, corn oil group; Ig, fish oil group; Cg_pre, corn oil group at baseline; Cg_aft, corn oil group at the end; Ig_pre, fish oil group at baseline; Ig_aft, fish oil group at the end.

The alpha diversity of gut fungal communities also revealed that no significant differences were found for the Chao1, Shannon, Simpson, and Pielou-e estimator between two groups (Figure 4A,B). In addition, the effect of fish oil supplementation on β-diversity was also limited and did not show statistical differences between the two groups (Figure 4C,D).

**Figure 5:**
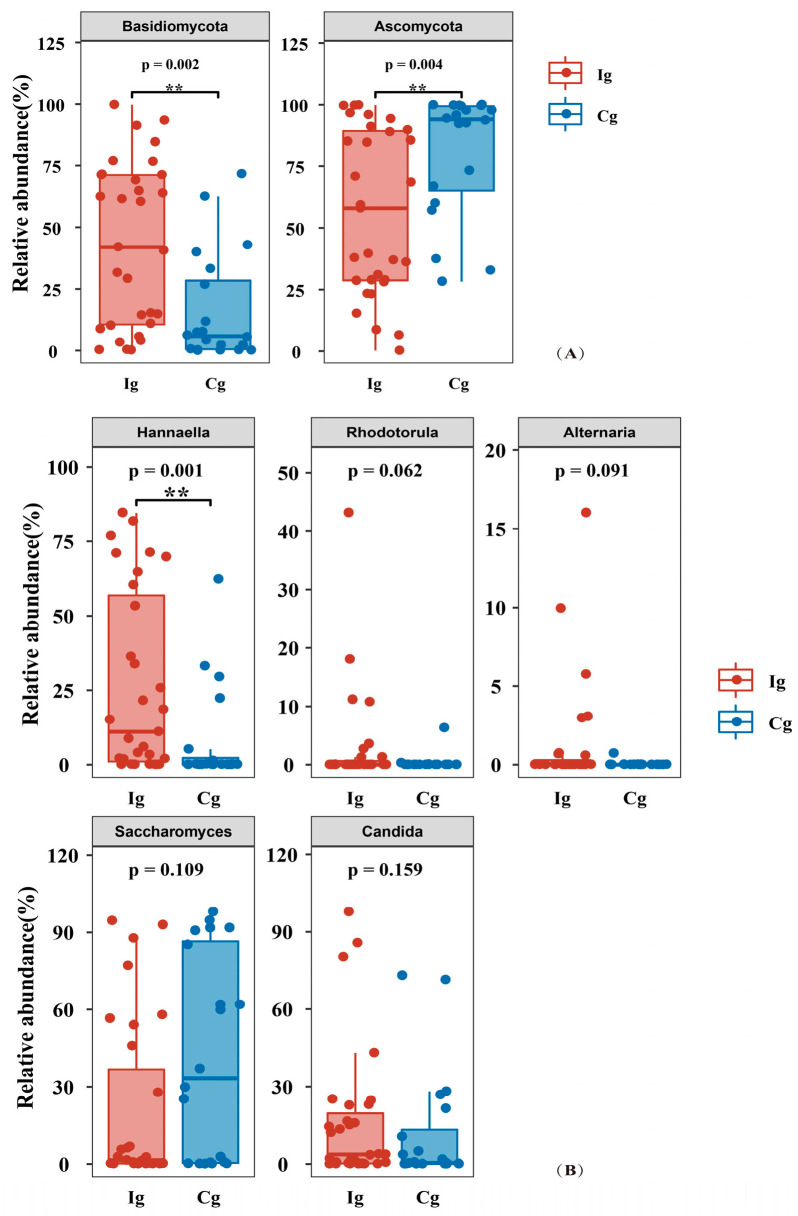
Changes in the relative abundance of gut fungi after intervention. (**A**) Phylum level of gut fungi; (**B**) genus level of gut fungi. ** *p* < 0.01; Cg, corn oil group; Ig, fish oil group. FDR < 0.2 was considered as significant.

For intestinal fungi, the intervention group at the endpoint and at the baseline showed changes in fungal species at the phylum, mainly an increase in the relative abundance of *Basidiomycota* and a decrease in the relative abundance of *Ascomycota* (Figure 4E). Figure 5A,B show changes in the relative abundance of gut fungi after intervention at the phylum and genus levels. At the level of the intestinal fungal phylum, compared with the corn oil control group, the relative abundance of *Ascomycota* was significantly lower (*p* = 0.015), and *Basidiomycota* was significantly higher (*p* = 0.008) in the fish oil group after three months. At the level of intestinal fungal genera, compared with the corn oil control group, the relative abundance of *Hannaella* was significantly higher in the fish oil group at the third month (*p* < 0.01).

### 3.4. Impact of Omega-3 PUFAs Intervention on Serum Lipid Metabolites

The results of main lipid metabolites after fish oil intervention are shown in Table 4. Compared with the corn oil group, the fish oil group showed a significant reduction in glycerophospholipid metabolites, including eight kinds. Specifically, fish oil-derived omega-3 fatty acids can significantly reduce LPC(22:4), LPE(22:4), PC(16:0/22:4), PC(18:1/22:4), PE(16:0/22:4), PE(O-16:0/22:4), PE(P-16:0/22:4), and PE(P-18:0/22:4). Figure S1 showed the results of the lipid composition analysis. The major classes of lipids detected in the samples can be divided into fatty acids, glycerolipids, glycerophospholipids, sphingolipids, and sterol lipids. The specific lipid subclasses are divided into FFA, DAG, TAG, LPC, LPE, PC, PE, CER, DCER, HCER, LCER, SM, and CE (Figure S1a). Lipids such as TAG, PC, and PE were significantly lower at the endpoint of the intervention group than the endpoint of the corn oil control group (Figure S1b). The results for lipid subclasses are presented as a circular graph, with TAG accounting for the highest percentage of 57.99%, followed by PE at 12.02% and PC at 8.45%. From the major lipid categories, glycerol lipids accounted for the highest percentage of 63.8%, followed by glycerophospholipids at 24.57% (Figure S1c). Circular plots are shown for lipid subclasses with significant differences in endpoints between the intervention group and the corn oil control group (Figure S1d). The highest percentage of TAG was 32.1%. The percentage of PE was 28.4%. The percentage of PC was 17.28%. Regarding lipid subgroups, the highest percentage of significantly changed lipid subclasses was glycerophospholipids (56.79%), followed by glycerolipids (38.27%). The KEGG pathway analysis mainly identified significant enrichment with the glycerophospholipid metabolism pathway (Figure S2).

### 3.5. Correlation Analyses

**Figure 6:**
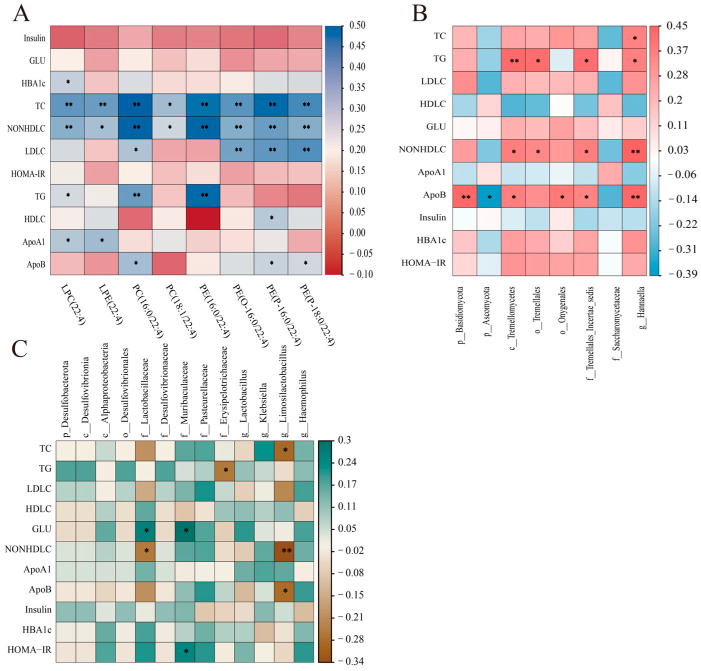
Heatmap of the correlation analysis. (**A**) Serum metabolites versus clinical biochemicals; (**B**) intestinal fungi versus clinical biochemicals; (**C**) intestinal bacteria versus clinical biochemicals. Spearman correlations were used. * *p* < 0.05, ** *p* < 0.01.

To further explore the relationship between serum lipid metabolites, intestinal bacteria, intestinal fungi, and clinical biochemical indicators, correlations were explored using a Spearman analysis. Biochemical parameters, including blood glucose and lipid metabolism, demonstrated significant correlations with most serum lipid metabolites. Total cholesterol and non-HDL cholesterol were significantly positively correlated with differential serum lipid metabolites. Triglycerides showed significant positive correlations with LPC(22:4), PC(16:0/22:4), and PE(16:0/22:4), while glycated hemoglobin also exhibited a significant positive correlation. Additionally, lipoprotein A1 and lipoprotein B were significantly positively correlated with certain serum lipid metabolites (Figure 6A).

For intestinal fungi, triglycerides, non-HDL cholesterol, and lipoprotein B showed significant positive correlations with certain intestinal fungi (Figure 6B). Additionally, a correlation analysis between gut bacteria and biochemical indicators revealed that total cholesterol was significantly negatively correlated with *g__Limosilactobacillus*. Blood glucose was significantly positively correlated with *f__Lactobacillaceae* and *f__Muribaculaceae*. Furthermore, non-HDL cholesterol, lipoprotein B, and HOMA-IR were significantly positively correlated with some gut bacteria (Figure 6C).

## 4. Discussion

Our findings revealed that the administration of fish oil supplements led to alterations in serum lipid levels, and no significant changes were observed in blood glucose levels. Also, it had an impact on the relative abundance of specific intestinal flora in individuals with type 2 diabetes. In particular, the intake of fish oil led to changes in glycerophospholipid metabolites in individuals diagnosed with type 2 diabetes. Fish oil-derived omega-3 PUFAs intervention resulted in a significant reduction in the relative abundance of gut bacteria, including *Colidextribacter*, *Ralstonia*, and *Klebsiella* genus. Omega-3 PUFAs intervention increased the relative abundance of *Limosilactobacillus*, *Lactobacillus*, and *Haemophilus* genii. Furthermore, there was a significant decrease in the relative abundance of gut fungi, specifically *Ascomycota*, and a significant increase in *Basidiomycota*. The results indicated that fish oil-derived omega-3 fatty acids exerted potential benefits in improving the metabolism of type 2 diabetes.

The results of this study suggest that patients with type 2 diabetes may benefit from fish oil supplementation. Significant reductions in serum fasting blood glucose, glycosylated hemoglobin levels, HOMA-IR, total cholesterol, triglyceride, LDL cholesterol, and non-HDL levels were observed in the fish oil group compared with the corn oil group after three months of intervention. Changes in the content of lipid metabolites were also observed. Most glycerophospholipid metabolites decreased in the fish oil group as compared with the placebo group. The major lipids in cell membranes are glycophospholipids (GPLs), of which phosphatidylcholines (PCs) and phosphatidylethanolamines (PEs) comprise more than half of the composition. Studies in animals suggest that disorders of PCs and/or PEs and their ratios may contribute to many risk factors for diabetes, such as insulin resistance [19,20]. It has been demonstrated that a Mediterranean diet can change circulating GPL profiles as well as concentrations of specific lipid metabolites [21,22,23,24]. These findings highlight the effect of diet on glycerophospholipid metabolites. In addition, compared with lipid metabolism parameters, the role of fish oil supplementation in improving glucose metabolism in type 2 diabetes mellitus is more limited, and further research is still needed to investigate.

The perturbation of glycerophospholipids (GPLs) is associated with the pathogenesis of diabetes mellitus. A prospective study among Chinese community residents indicated that especially phosphatidylcholines associated with the de novo lipogenesis (DNL) pathway were positively associated with incident diabetes [25]. Also, findings from a previous Chinese nested case-control study showed that different GPL genes, such as LPC(16:1) and PE(P-18:0/20:4), had significant impacts on diabetes prevalence [26]. In addition, animal experiments demonstrate that inhibition of PC biosynthesis by a choline-deficient diet or deletion of PE *N*-methyltransferase improves insulin resistance, fasting glucose levels, and weight gain in mice fed a high-fat diet and may partially support the observed association of GPL with diabetes [19,20,27]. Results from the prospective study showed that four diabetes-related GPLs, including PC(16:0/16:1), PC(16:0/18:1), PC(18:0/16:1), and PE (16:0/16:1), were associated with unhealthy dietary patterns [25]. We found that fish oil-derived omega-3 fatty acids can significantly reduce LPC(22:4), LPE(22:4), PC(16:0/22:4), PC(18:1/22:4), PE(16:0/22:4),PE(O-16:0/22:4), PE(P-16:0/22:4), and PE(P-18:0/22:4). These findings suggest the potential benefit of fish oil in regulating diabetes-related lipid metabolites. The EPIC-Potsdam study found that low fish intake was associated with monounsaturated PCs such as PC(34:1) [28]. A high-carbohydrate diet may upregulate DNL, resulting in its unique fatty acyl chain, which may be an abundant substrate for GPL synthesis [29]. An early cohort study showed that erythrocyte DNL fatty acids were associated with a high carbohydrate/fat ratio and an increased incidence of diabetes mellitus [30]. There is little clear explanation as to what causes high levels of DNL fatty acids to lead to diabetes, but animal studies suggest that DNL fatty acids are involved in endoplasmic reticulum stress, endothelial dysfunction, and inflammatory responses [31,32]. Specific structures in the GPL may reflect specific dietary exposures that can link specific metabolic pathway(s) to diabetes risk. These results suggest that reducing the risk of diabetes by altering glycerophospholipid metabolites may be a potential target.

The intestinal microbiota represents a complex community involving bacteria in the gastrointestinal tract that usually maintain a reciprocal relationship with their hosts. Numerous studies have shown that the pathogenesis of T2DM is closely related to intestinal flora [33,34]. Dietary factors play a crucial role in the development and progression of T2DM. One of the most common dietary approaches for T2DM is to increase the intake of omega-3 PUFAs [35,36]. Dysbiosis of the intestinal flora can promote the entry of Lipopolysaccharide (LPS) into systemic circulation by increasing intestinal permeability, leading to inflammation and metabolic dysfunction. In the present study, after fish oil intervention, the *Firmicutes*/*Bacteroidetes* (F/B) ratio tended to increase, and no significant changes were observed in the relative abundance of *Bacteroidetes* and *Proteobacteria*. Firmicutes and Bacteroidetes are generally involved in the regulation of lipid and bile acid metabolism and in the maintenance of host energy balance [37]. The *Firmicutes* phylum is involved in the degradation of oligosaccharides, fibers, and starches, helping the host intestine absorb energy from food. Moreover, *Firmicutes* can produce volatile fatty acids [38]. In the gut, *Bacteroidetes* perform a variety of functions, including the degradation of carbohydrates as well as the production of butyrate [39]. The *Firmicutes* phylum increases the nutrients that are available to the host, whereas the *Bacteroidetes* phylum is less energetically advantageous [40]. The *Firmicutes*/*Bacteroidetes* (F/B) ratio is a microbial marker of intestinal dysbiosis. The proliferation of *Proteobacteria* has resulted in a disruption of the intestinal microbiota, subsequently inducing inflammation within the intestines [41]. According to reports, there is a general reduction in the abundance of *Bacteroidetes* in individuals who are obese [42]. The absence of this change in the current study may be attributed to the potential influence of confounding variables on the intervention trial, as determined through our analysis. After fish oil intervention, the phylum of *Desulfobacterota* was significantly reduced at the phylum level in diabetic patients. *Desulfobacterota* phylum can reduce sulfur compounds through the pathway of sulfite reductase and degrade butyrate-producing microorganisms, playing a crucial role in energy metabolism [39]. In addition, at the level of the fungal phylum, we also found a significant reduction in the fungal Ascomycota phylum. The phylum of *Ascomycota* may be associated with low-grade inflammation [43]. The findings demonstrate that fish oil possesses the capability to exert anti-inflammatory properties and modulate the microbiota linked to inflammation, thereby improving the condition of disease.

At the genus level, Gram-negative bacteria such as *Colidextribacter*, *Ralstonia*, and *Klebsiella*, which are associated with inflammation and infection, were significantly reduced after fish oil intervention, suggesting that fish oil intervention improves the anti-inflammatory capacity of diabetes. *Limosilactobacillus* and *Lactobacillus*, as Gram-positive bacteria, were significantly elevated after fish oil intervention. It has been shown that oral administration of *Lactobacillus* significantly improved epithelial barrier function and consequently reduced inflammatory cytokines, preventing to some extent liver and colonic tissue damage. In addition, *Lactobacillus acidophilus* treatment modulates expressed genes related to glucose and lipid metabolism [44,45,46]. Omega-3 polyunsaturated fatty acids can reverse gut microbial dysbiosis by increasing the number of beneficial bacterial species, including *Lactobacillus*, *Bifidobacterium*, and butyrate-producing bacteria [47]. The findings of a randomized controlled trial demonstrated that the intake of omega-3 fatty acids led to a notable augmentation in the abundance of *Lactobacillus* and *lactobacilli* within the gastrointestinal tract of middle-aged individuals who were in good health [48]. This finding aligns closely with the results obtained in our study. The present findings suggest that fish oil may improve metabolism and, ultimately, diabetes-related symptoms by increasing *Lactobacillus* in the intestinal flora and may influence glycerophospholipid metabolic pathways, thus improving diabetes-related symptoms. In addition, fish oil may also increase the abundance of beneficial intestinal bacteria and decrease the abundance of harmful intestinal bacteria by modulating intestinal flora, which may ultimately lead to diabetes improvement. The specific mechanism by which fish oil affects the glycerophospholipid metabolic pathway and ultimately affects diabetes still needs to be further explored and verified.

Our study has certain advantages. First, the present study employed a double-blind, placebo-controlled design to ensure rigorous quality control of the trial data from the population while also minimizing the influence of confounding factors. In addition, our research employs various technologies, including 16S rDNA, ITS high-throughput sequencing, and untargeted lipidomics to investigate the potential impact of fish oil supplementation on serum lipid metabolites as well as intestinal bacteria and fungi in individuals with T2DM. Of course, this study is subject to certain limitations, primarily stemming from the restricted sample size of the current study. Consequently, additional validation through large-scale population trials is imperative. In addition, despite the stringent control of the population, the presence of uncontrolled confounding factors persists, potentially exerting an influence on the study outcomes.

## 5. Conclusions

In summary, fish oil-derived omega-3 PUFAs can affect the structure and function of the intestinal microbiota in type 2 diabetic patients. The observed decrease in pathogenic bacterial species and the enhancement of beneficial species may have significant implications for gut health and systemic inflammation, both of which are pivotal in the management of diabetes. Further research is warranted to comprehensively elucidate these microbiota alterations’ long-term benefits and underlying mechanisms.

## Figures and Tables

**Table 1:** Baseline clinical and demographic characteristics.

	All (*n* = 101)	Corn Oil (*n* = 49)	Fish Oil (*n* = 52)	*p* Value
Age (years)	53.81 ± 8.82	54.29 ± 8.45	53.37 ± 9.22	0.603
Sex (male/female)	66/39	35/14	31/21	0.212
Duration of T2DM (years)	6.0 (3.0, 11.0)	6.0 (2.0, 11.0)	7.0 (3.0, 12.0)	0.758
BMI (kg/m^2^)	24.95 ± 2.94	24.86 ± 2.86	25.03 ± 3.04	0.767
Waist-to-hip ratio	0.91 ± 0.06	0.90 ± 0.06	0.91 ± 0.06	0.478
Diurnal systolic BP (mm Hg)	129.90 ± 17.52	129.53 ± 18.02	130.25 ± 17.20	0.838
Diurnal diastolic BP (mm Hg)	76.90 ± 10.46	76.37 ± 10.53	77.4 ± 10.47	0.621
Fasting plasma glucose (mmol/L)	6.38 ± 1.78	6.25 ± 2.00	6.49 ± 1.56	0.502
Hemoglobin A1c (mmol/moL)	51.56 ± 11.01	52.54 ± 11.98	50.63 ± 10.04	0.387
Fasting plasma insulin (mU/L)	10.76 ± 8.89	10.92 ± 11.17	10.61 ± 6.13	0.861
HOMA-IR	1.98 ± 0.76	1.98 ± 0.86	1.98 ± 0.65	0.961
Total cholesterol (mmol/L)	4.69 ± 0.90	4.56 ± 0.92	4.81 ± 0.87	0.160
LDL-C (mmol/L)	2.40 ± 0.81	2.49 ± 0.87	2.32 ± 0.75	0.302
HDL-C (mmol/L)	1.25 ± 0.30	1.30 ± 0.27	1.19 ± 0.33	0.109
Triglycerides (mmol/L)	2.28 ± 2.09	2.09 ± 1.74	2.46 ± 2.37	0.370
ApoA1 (g/L)	1.14 ± 0.23	1.17 ± 0.22	1.11 ± 0.24	0.159
ApoB (g/L)	0.80 ± 0.17	0.83 ± 0.18	0.77 ± 0.15	0.084
Total physical activity (MET-minute/per week)	2807.52 ± 509.28	2824.03 ± 552.39	2791.97 ± 469.95	0.754
Diabetes medications				
Oral antidiabetic agent (s)	40	19	21	
Insulin injection	19	9	10	0.658
Both	35	19	16	
Dietary therapy alone	7	2	5	

**Table 2:** Comparison of the dietary intake between study groups at baseline and at the end of the intervention.

Variable	Corn Oil Group (*n* = 49)	Fish Oil Group (*n* = 52)	MD (95% CI), *p*
Energy (kcal/day)			
Baseline	1653.75 ± 325.61	1796.40 ± 537.18	−142.65 (−323.35, 38.04), 0.120
3 months	1695.73 ± 457.55	1808.11 ± 533.41	−112.39 (−316.84, 92.06), 0.278
MD (95% CI), *p*	41.98 (−119.28, 203.23), 0.602	11.71 (−193.58, 217.01), 0.909	
Fat (g)			
Baseline	51.22 ± 13.00	58.46 ± 24.12	−7.24 (−15.28, 0.80), 0.077
3 months	52.72 ± 10.11	59.95 ± 22.93	−7.23 (−14.60, 0.14), 0.054
MD (95% CI), *p*	1.50 (−0.99, 3.99), 0.230	1.49 (−2.77, 5.76), 0.485	
Protein (g)			
Baseline	51.78 ± 15.66	53.31 ± 17.43	−1.54 (−8.35, 5.28), 0.655
3 months	51.88 ± 16.69	52.61 ± 18.82	−0.73 (−8.05, 6.58), 0.842
MD (95% CI), *p*	0.10 (−7.37, 7.57), 0.978	−0.70 (−8.23, 6.83), 0.853	
Carbohydrate (g)			
Baseline	246.42 ± 63.95	264.26 ± 89.32	−17.85 (−49.92, 14.23), 0.272
3 months	253.43 ± 98.89	264.53 ± 99.75	−11.10 (−51.83, 29.64), 0.590
MD (95% CI), *p*	7.02 (−26.68, 40.71), 0.677	0.27 (−42.13, 42.66), 0.990	
Cellulose (g)			
Baseline	7.50 ± 5.32	7.51 ± 4.52	−0.01 (−2.03, 2.00), 0.990
3 months	7.10 ± 3.40	7.04 ± 3.20	0.06 (−1.29, 1.41), 0.930
MD (95% CI), *p*	−0.39 (−2.22, 1.43), 0.666	−0.47 (−2.10, 1.17), 0.568	

**Table 3:** Comparison of the glucose parameters and lipid profile between study groups at baseline and at the end of the intervention.

Variable	Corn Oil Group (*n* = 49)	Fish Oil Group (*n* = 52)	MD (95% CI), *p*
FBS (mmol/L)			
Baseline	6.25 ± 2.00	6.49 ± 1.56	−0.24 (−0.95, 0.47), 0.502
3 months	7.66 ± 2.61	6.76 ± 1.55	0.90 (0.05, 1.75), 0.039
MD (95% CI), *p*	1.41 (0.78, 2.04), <0.001	0.27 (−0.27, 0.81), 0.328	
FI (mU/L)			
Baseline	10.92 ± 11.17	10.61 ± 16.13	0.31 (−3.22, 3.84), 0.861
3 months	10.64 ± 6.95	8.87 ± 2.81	1.77 (−0.36, 3.90), 0.102
MD (95% CI), *p*	−0.29 (−2.58, 2.00), 0.801	−1.74 (−3.20, −0.29), 0.019	
HbA1c (%)			
Baseline	6.96 ± 1.10	6.78 ± 0.92	0.17 (−0.22, 0.57), 0.387
3 months	7.03 ± 1.04	6.66 ± 0.81	0.37 (0.00, 0.74), 0.048
MD (95% CI), *p*	0.07 (−0.21, 0.36), 0.606	−0.12 (−0.33, 0.09), 0.259	
HOMA-IR			
Baseline	1.98 ± 0.86	1.98 ± 0.65	0.00 (−0.31, 0.29), 0.961
3 months	2.48 ± 1.24	2.02 ± 0.62	0.46 (0.07, 0.85), 0.022
MD (95% CI), *p*	0.51 (0.23, 0.79), 0.001	0.04 (−0.17, 0.25), 0.705	
TC (mmol/L)			
Baseline	4.56 ± 0.92	4.81 ± 0.87	−0.25 (−0.61, 0.10), 0.160
3 months	5.06 ± 1.02	4.32 ± 0.75	0.73 (0.38, 1.08), <0.001
MD (95% CI), *p*	0.50 (0.28, 0.71), <0.001	−0.49 (−0.71, −0.27), <0.001	
TG (mmol/L)			
Baseline	2.09 ± 1.74	2.46 ± 2.37	−0.37 (−1.20, 0.45), 0.370
3 months	2.06 ± 2.26	1.33 ± 0.63	0.73 (0.06, 1.40), 0.034
MD (95% CI), *p*	−0.03 (−0.37, 0.31), 0.867	−1.13 (−1.75, −0.51), 0.001	
LDL-C (mmol/L)			
Baseline	2.49 ± 0.87	2.32 ± 0.75	0.17 (−0.15, 0.49), 0.302
3 months	2.89 ± 0.89	2.58 ± 0.65	0.31 (0.00, 0.61), 0.048
MD (95% CI), *p*	0.40 (0.21, 0.59), <0.001	0.26 (0.05, 0.47), 0.018	
HDL-C (mmol/L)			
Baseline	1.30 ± 0.27	1.19 ± 0.33	0.10 (−0.01, 0.22), 0.084
3 months	1.55 ± 0.32	1.46 ± 0.36	0.09 (−0.05, 0.22), 0.210
MD (95% CI), *p*	0.25 (0.19, 0.30), <0.001	0.27 (0.21, 0.32), <0.001	
Non-HDL (mmol/L)			
Baseline	3.26 ± 0.86	3.62 ± 0.92	−0.36 (−0.71, 0.00), 0.046
3 months	3.51 ± 1.10	2.86 ± 0.78	−0.36 (−0.71, 0.00), 0.046
MD (95% CI), *p*	0.24 (0.01, 0.48), 0.041	−0.76 (−0.99, −0.52), <0.001	0.65 (0.26, 1.03), 0.001

**Table 4:** Statistically significant differences in the change in lipid metabolites for type 2 diabetes between the fish oil group and corn oil group.

	Fish Oil Group (*n* = 38)	Corn Oil Group (*n* = 37)
LPC(22:4)	28.15 (23.42, 38.51)	61.33 (49.05, 77.03)
LPE(22:4)	1.46 (0.88, 2.20)	3.08 (2.25, 4.43)
PC(16:0/22:4)	1869.97 (1468.17, 2428.25)	3926.66 (3054.74, 5474.43)
PC(18:1/22:4)	163.13 (133.72, 186.76)	335.58 (274.94, 455.75)
PE(16:0/22:4)	8.31 (6.00, 15.26)	28.48 (19.81, 42.54)
PE(O-16:0/22:4)	13.64 (8.92, 20.33)	33.35 (24.75, 42.23)
PE(P-16:0/22:4)	78.23 (57.80, 88.45)	156.51 (115.55, 194.56)
PE(P-18:0/22:4)	84.54 (57.73, 107.74)	176.93 (125.70, 211.15)

Data are expressed as median (P25, P75). LPC(22:4): lysophosphatidylcholine(22:4); LPE(22:4): lysophosphatidylethanolamine(22:4); PC(16:0/22:4): phosphatidylcholine(16:0/22:4); PC(18:1/22:4): phosphatidylcholine(18:1/22:4); PE(16:0/22:4): phosphatidylethanolamine(16:0/22:4); PE(O-16:0/22:4): plasmenylethanolamine(O-16:0/22:4); PE(P-16:0/22:4): plasmanylphosphatidylethanolamine(P-16:0/22:4); PE(P-18:0/22:4): plasmanylphosphatidylethanolamine(P-18:0/22:4).

## Data Availability

All data that support the findings of this study are available from the corresponding author upon reasonable request, but they are not publicly accessible for ethical reasons.

## Supplementary Materials

The following supporting information can be downloaded at: https://www.mdpi.com/article/10.3390/nu16213755/s1, Figure S1: Lipid composition analysis; Figure S2: KEGG pathway analysis.
