# Supplementary material for: Improvement in Glycolipid Metabolism Parameters After Supplementing Fish Oil-Derived Omega-3 Fatty Acids Is Associated with Gut Microbiota and Lipid Metabolites in Type 2 Diabetes Mellitus
Source: Nutrients. 2024 Oct 31;16(21):3755. doi: 10.3390/nu16213755 (PMC11547733; doi:10.3390/nu16213755)
Supplement: Supplementary file 1 [file nutrients-16-03755-s001.zip › nutrients-3269108-supplementary.pdf]

Supplementary

Supplementary 1. Lipid composition analysis

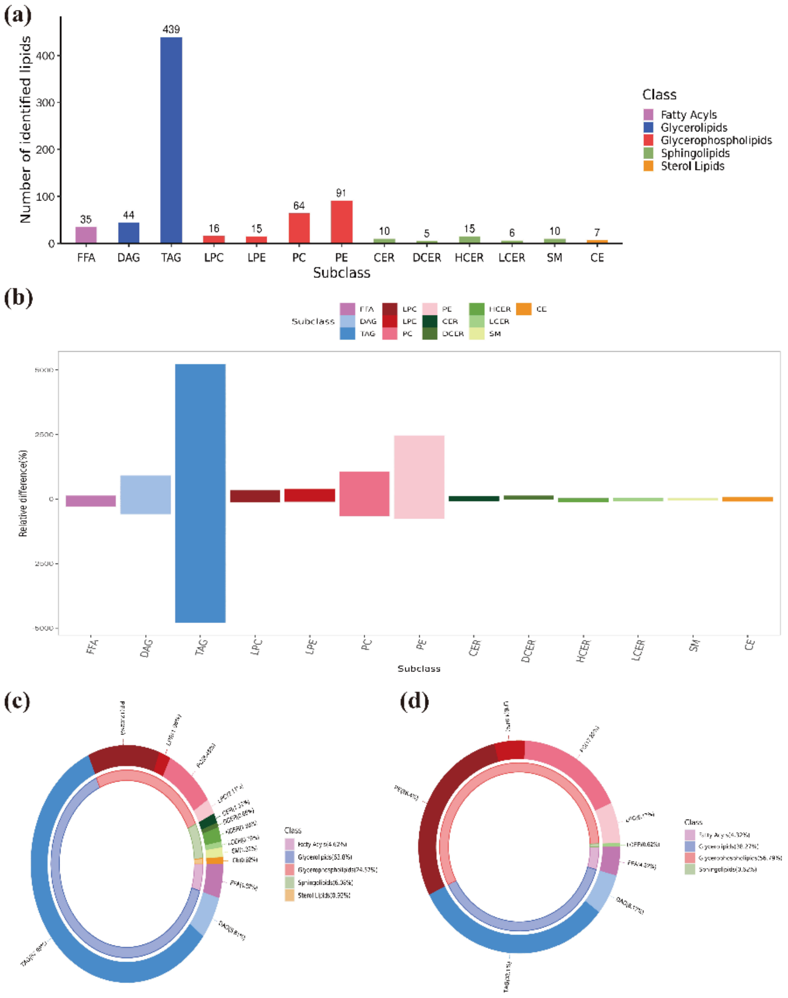

Figure S1. Lipid composition analysis. (a) Lipid subclasses detected in the samples and the amount of lipids in each subclass; (b) Bar graph of the change in lipid content in the control endpoint group versus the intervention endpoint group; Each bar in the bar graph represents a class of metabolites, and the vertical coordinates of the graph represent the relative percentage change in the content of each substance for that group of comparison. The horizontal coordinates of the bar graph of lipid groups indicate the classification information of lipids. (c) Circular plot of lipid distribution in the sample; (d) Loop plot of lipid composition in the control endpoint group versus the intervention endpoint group.

Supplementary 2. KEGG pathway analysis

(a)

Pathway	Description	# compounds (dem)	Compounds (dem)	# compounds (all)	Compounds (all)	Total
hsa00564	Glycerophospholipid metabolism - Homo sapiens (human)	3	C04230;C00157;C00350	3	C04230;C00157;C00350	56
hsa01100	Metabolic pathways - Homo sapiens (human)	3	C00157;C00350;C00422	3	C00157;C00350;C00422	2966
hsa04723	Retrograde endocannabinoid signaling - Homo sapiens (human)	2	C00157;C00350	3	C00165;C00157;C00350	19
hsa05231	Choline metabolism in cancer - Homo sapiens (human)	2	C04230;C00157	3	C00165;C04230;C00157	11

(b)

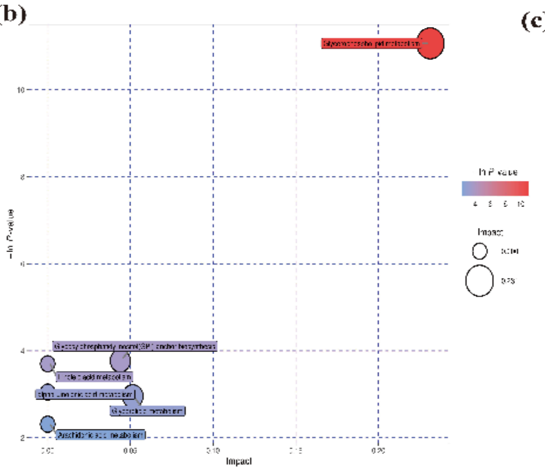

(c)

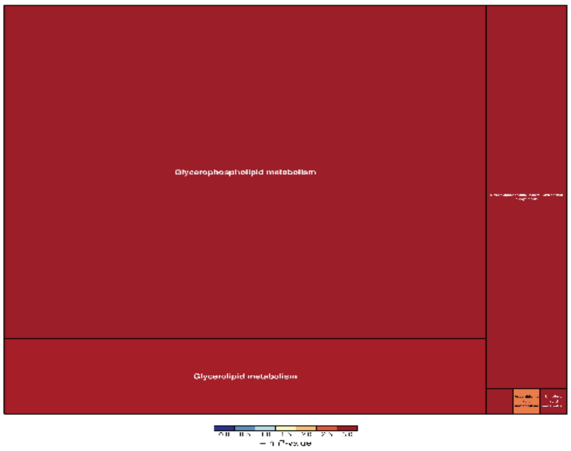

Figure S2. KEGG pathway analysis. (a) KEGG analysis; pathway represents the KEGG PATHWAY database ID of the pathway; Description represents the name of the pathway; # compounds (dem) represents the number of differential metabolites within the pathway; Compounds (dem) represents the KEGG COMPOUND database ID of the differential metabolites within the pathway; # compounds (all) represents the number of all metabolites detected in the pathway; Compounds (all) represents the KEGG COMPOUND database ID of all metabolites detected in the pathway; Total represents the number of all metabolites in the pathway. (b) pathway analysis, Bubble plot; (c)pathway analysis, tree map.
